# HuR interacts with human immunodeficiency virus type 1 reverse transcriptase, and modulates reverse transcription in infected cells

**DOI:** 10.1186/1742-4690-5-47

**Published:** 2008-06-10

**Authors:** Julie Lemay, Priscilla Maidou-Peindara, Thomas Bader, Eric Ennifar, Jean-Christophe Rain, Richard Benarous, Lang Xia Liu

**Affiliations:** 1 Institut Cochin, Université Paris Descartes, CNRS (UMR8104), Paris, France; 2 Inserm, U567, Paris, France; 3 Architecture et réactivité de l'ARN, UPR 9002 CNRS, 15 rue René Descartes, 67084 Strasbourg, France; 4 Hybrigenics S.A., F-75014 Paris, France; 5 current address : University Children's Hospital, Division of Immunology, Steinwiesstrasse 75, CH-8032, Zürich, Switzerland; 6 current address : CellVir, 4 rue Pierre Fontaine, 9100 Evry, France; 7 Current Address: Institutes of Life and Health Engineering, Jinan University, 601 Huang Pu Avenue West, Guangzhou 510632, China.

## Abstract

Reverse transcription of the genetic material of human immunodeficiency virus type 1 (HIV-1) is a critical step in the replication cycle of this virus. This process, catalyzed by reverse transcriptase (RT), is well characterized at the biochemical level. However, in infected cells, reverse transcription occurs in a multiprotein complex – the reverse transcription complex (RTC) – consisting of viral genomic RNA associated with viral proteins (including RT) and, presumably, as yet uncharacterized cellular proteins. Very little is known about the cellular proteins interacting with the RTC, and with reverse transcriptase in particular. We report here that HIV-1 reverse transcription is affected by the levels of a nucleocytoplasmic shuttling protein – the RNA-binding protein HuR. A direct protein-protein interaction between RT and HuR was observed in a yeast two-hybrid screen and confirmed *in vitro *by homogenous time-resolved fluorescence (HTRF). We mapped the domain interacting with HuR to the RNAse H domain of RT, and the binding domain for RT to the C-terminus of HuR, partially overlapping the third RRM RNA-binding domain of HuR. HuR silencing with specific siRNAs greatly impaired early and late steps of reverse transcription, significantly inhibiting HIV-1 infection. Moreover, by mutagenesis and immunoprecipitation studies, we could not detect the binding of HuR to the viral RNA. These results suggest that HuR may be involved in and may modulate the reverse transcription reaction of HIV-1, by an as yet unknown mechanism involving a protein-protein interaction with HIV-1 RT.

## Introduction

HIV-1 reverse transcriptase (RT) is a DNA- and RNA-dependent DNA polymerase responsible for converting the virion ssRNA genome into a dsDNA genome once the virus has entered the cell [1]. HIV-1 RT also displays RNA degradation activity (RNase H), independent of its polymerase activities. Both activities are essential for the reverse transcription process *in vivo*.

HIV-1 reverse transcriptase is incorporated into virions, during their assembly, as part of the Gag-Pol precursor. It is processed into two subunits by the viral protease, during particle maturation, after budding. The p66 subunit includes domains responsible for the RNase H and DNA polymerase activities, whereas the p51 subunit bears only the polymerase domain. The two subunits dimerize within the viral particle, and form the p66/p51 heterodimer, the active form of the enzyme [2]. Reverse transcription occurs essentially in the cytoplasm once the virus has entered the cell. It is mediated by a complex formed by two copies of the viral RNA, associated viral proteins, including RT, and, presumably, cellular proteins that have yet to be characterized. This reverse transcription complex (RTC) is gradually transformed into the preintegration complex (PIC), during its progressive migration to the nucleus. The PIC is responsible for ensuring the integration of the proviral genomic DNA generated by reverse transcription into the host genome (recently reviewed in [3]).

Recent studies point towards the importance of cellular co-factors for an efficient reverse transcription of HIV-1 *in vivo *[4,5]. However, the cellular factors involved in this reaction have not yet been identified. Moreover, there have been very few reports of cellular proteins interacting with HIV-1 RT. Hottiger *et al*. showed that the HIV-1 p66 monomer interacts directly with beta-actin [6]. Olova *et al*. have shown that eRF1 interacts directly with the reverse transcriptase of the murine retrovirus, M-MuLV [7], but not with HIV-1 RT. We searched for other molecules potentially interacting with HIV-1 RT, by carrying out yeast two-hybrid screening with HIV-1 p66 as the bait and a CEMC7 cell line cDNA library as the prey. We identified HuR (or ELAVL1) as potentially interacting with HIV-1 RT.

HuR is a ubiquitous protein involved essentially in stabilizing mRNAs by binding to adenylate/uridylate-rich elements (AREs). HuR is mostly found in the nucleus, but can shuttle to the cytoplasm, and has also been found associated with stress granules [8,9]. There is a direct correlation between the capacity of HuR to stabilize mRNA and its shuttling to the cytoplasm. HuR shuttling can be observed in the HIV cell targets, T lymphocytes, following their activation, by the binding of ICAM-1 to the LFA-1 integrin, for example [10]. Furthermore, HuR levels vary during the cell cycle and are maximal during the G2 phase [11,12].

We show here that HuR interacts with HIV-1 RT in the RNase H region, and that HuR silencing, using specific siRNAs, or overexpression, through the transient transfection of an HuR expression vector, greatly affects the reverse transcription process.

## Materials and methods

### Yeast two-hybrid screening

Two-hybrid screens were carried out with a cell-to-cell mating protocol, as previously described [13,14]. Random cDNA librairies from CEMC7 cells were constructed into the pP6 plasmid derived from the original pACT2, by blunt-end ligation of an *Sfi*I linker. *E. coli *DH10B (Invitrogen, Carlsbad, California) was transformed with these libraries, giving over 50 million clones. *S. cerevisiae *was transformed with these libraries, by the classical lithium acetate protocol. Ten million independent colonies were collected, pooled, and stored at -80°C as aliquots of the same library. The HIV-1 reverse transcriptase gene was amplified with appropriate primers from the YU2 proviral DNA plasmid and inserted into pB27 [15]. For the rebound screening, HuR was inserted into pB27, using appropriate primers, and the HIV genomic library used was as previously described [13,15].

### Plasmids

The prokaryotic expression vector, p6H-RT-PR, was kindly provided by Dr Giovanni Maga and has been described elsewhere [16]. GST-HuR was constructed by PCR amplification of the HuR gene from the image clone # IMGCLO2901220 (accession # BC003376) bought from GeneService (Cambridge, UK), using the following primers: sense: 5'-GCG GCG GAA TTC TCT AAT GGT TAT GAA GAC CAC A-3', antisense: 5'-GCG GCG GTC GAC TTA TTT GTG GGA CTT GTT GG-3'. The resulting fragment was inserted between the *Eco*RI and *Sal*I sites of pGEX4T1 (GE healthcare). pCMV-HuR was constructed by introducing this fragment into pcDNA3 (Invitrogen). pNL4-3AREmut was generated by site-directed mutagenesis on pNL4-3 [17], using the "overlap extension PCR" method with *pfu *polymerase (Stratagene), as described elsewhere [18]. The following primers were used: sense: 5'-CAC TAC TTC GAC TGC TTC TCC GAG TCT GCT ATA AGA AAT ACC ATA TTA GGA CGT AT-3', antisense: 5'-AGA CTC GGA GAA GCA GTC GAA GTA GTG CAG ATG AAT TAG TTG GTC TGC-3'. The Flag-p66 construct was generated by PCR amplification of the HIV-1 NL4-3 p66 region and its insertion into the pSG5 vector (Stratagene).

### Production and purification of recombinant proteins

6xHis-tagged RT was produced from *E. coli *DH5α transformed with the p6H-RT-PR expression vector. GST-HuR was produced from *E. coli *BL21 transformed with pGEX4T1-HuR. Overnight cultures of bacteria were diluted to an OD of 0.05 in LB media (50 μg/ml ampicillin) and cultured to an OD of 0.4. Then, 1 mM isopropyl-1-thio-β-D-galactopyranoside (IPTG) was added to the cultures, which were incubated for 3 hours to induce protein production. The His-RT bacterial pellet was weighed and ground for 2 minutes in a chilled mortar with 2.5 parts of type A-5 aluminum oxide (Sigma), at 4°C. The extract was then resuspended in extraction buffer (300 mM NaCl, 50 mM sodium phosphate) and centrifuged at 12,000 g for 20 minutes at 4°C. His-tagged recombinant proteins were purified from the supernatant, using BD-TALON IMAC Resin (Clontech), according to the manufacturer's instructions. The GST-HuR bacterial pellet was resuspended in lysis buffer (20 mM Tris-Cl pH 7.5, 2 mM DTT, 1 mM EDTA, 10% glycerol, 1 M NaCl, 1 μg/ml lysosyme, 100 μg/ml chloramphenicol, 0.1 mM PMSF) supplemented with protease inhibitor cocktail (Sigma), and subjected to 3 15-second sonication pulses, on ice. The lysate was centrifuged for 30 minutes, at 15,700 g and 4°C. The supernatant was incubated with Glutathione-Sepharose 4B beads (GE Healthcare) for 1 hour at 4°C. The beads were washed several times in lysis buffer and proteins were eluted in 20 mM reduced glutathione (Roche).

### HTRF assay

GST-HuR or GST was serially diluted in the following buffer: 50 mM phosphate buffer, 0.8 M potassium phosphate, 0.0075% Tween-20 and 2 mM MgCl_2_. RT-His was diluted in the same buffer such that the final reaction mixture contained 10 ng/ml. Anti-GST-TBPEu^3+ ^and anti-HisXL665 antibodies were reconstituted as recommended by the manufacturer. The proteins were incubated with both antibodies and readings were taken in a black 384 half-well plate (Greiner). The plate was read with the PHERAstar apparatus from BMG LABTEC at 665 nm (XL665 fluorescence) and 620 nm (europium cryptate fluorescence) after excitation at 337 nm. This dual measurement made it possible to calculate the signal ratio. The specific signal was obtained as follows:

Fluorescence ratio R = [signal 665 nm/signal 620 nm] × 10,000. ΔR = [R_sample _- R_negative_] and ΔF (%) = [ΔR/R_negative_] × 100.

### Cells, viruses, and transfections

HEK293T, HeLa, HeLa P4.2 and HeLa R7 Neo cells were grown in DMEM (Invitrogen) supplemented with 10% fetal calf serum (FCS; Invitrogen) and antibiotics (100 units/ml penicillin, 100 mg/ml streptomycin; Invitrogen). HeLa P4.2 (CD4+, LTR-*LacZ*) cells were cultured in the presence of 200 μg/ml G418 [19]. HeLa R7 Neo (stably infected with the HIV-1 neo Δ*env *virus) cells were cultured in the presence of 500 μg/ml G418, and were kindly provided by Dr. Pierre Sonigo [20]. Jurkat cells were grown in RPMI 1640 (Invitrogen), supplemented with 10% FCS and antibiotics (100 units/ml penicillin, 100 mg/ml streptomycin). For the overexpression and immunofluorescence assays, HeLa cells were tranfected with Fugene-6 reagent (Roche), according to the manufacturer's protocol. Virus stocks were generated by transfecting HEK293T cells with the provirus pNL4-3 or pNL4-3AREmut, using the calcium phosphate technique (Stratagene). Single round pseudotyped viruses were obtained by cotransfecting cells with pNL4-3Δenv and a VSV-G envelope expression vector, as previously described [21]. Viral particle production in the cell culture supernatant was evaluated with the anti-p24 ELISA kit from Beckman Coulter. Purified viral particles were obtained by passing the cell culture supernatant through a filter with 0.45 μM pores, and centrifuging the filtrate on a 20% sucrose cushion at 27,000 rpm for 90 minutes at 4°C in an SW28 rotor. For infected cell quantification, HeLa P4.2 cells were fixed in 0.5% glutaraldehyde (Sigma) in phosphate-buffered saline (PBS) and stained overnight at 4°C in 4 mM potassium ferrocyanide, 4 mM potassium ferricyanide, 2 mM MgCl_2 _and 400 μg/ml X-Gal (Roche) in PBS.

### siRNA assays

siRNA HuR1 (HuR1.1: GCCUGUUCAGCAGCAUUGGTT and HuR1.2: CCAAUGCUGCUGAACAGGCTT) was synthesized by Eurogentec and annealed according to the manufacturer's instructions. siRNA HuR2 and HuR3 were obtained from Qiagen (cat.no. SI00300139 and SI03246887 respectively). The negative control, a non targeting siRNA (siCONTROL) was obtained from Dharmacon. HeLa or HeLa P4.2 cells were transfected twice with 30 nM of siRNA, using Oligofectamine reagent (Invitrogen).

### Quantification of early and late RT products in infected HeLa cells

HeLa cells were transfected either twice with 30 nM siHuR1 (or siCtrl) siRNA during a 24-hour period, using Oligofectamine reagent (Invitrogen), or with 1 μg/mL pCMV-HuR (or the empty vector), using FUGENE-6 (Roche Applied Science). Cells were incubated for 24 hours and then washed three times with PBS and infected with NL4.3(ΔEnv) VSV-G-pseudotyped virus at a multiplicity of infection (MOI) of 0.1. About 16 hours after infection, cells were harvested, washed in PBS and treated with 500 units of DNase I (Roche Diagnostics) for 1 h at 37°C. Total DNA was then extracted, using a QIAamp blood DNA minikit (Qiagen), and early and late RT products (minus-strand stop DNA and full-length DNA, respectively) were quantified by real-time PCR. DNA samples were assayed in duplicate, using the LC FastStart DNA hybridization probes kit (Roche Diagnostics). Fluorescence was measured on a LightCycler^® ^2.0 Instrument (Roche Applied Science). The following primers and probes were used: early RT forward primer: 5'-TAACTAGGGAACCCACTG-3'; early RT reverse primer: 5'-CACTGACTAAAAGGGTCT-3'; early RT probe1: GCTTGCCTTGAGTGCTCA (Fluo); early RT probe2: (Red640) GTAGTGTGTGCCCGTCT (Phosphate); late RT forward primer: 5'-CGTCTGTTGTGTGACT-3'; late RT reverse primer: 5'-TTTTGGCGTACTCACC-3'; late RT probe1: ATCTCTCGACGCAGGAC (Fluo); late RT probe2: (Red640) GGCTTGCTGAAGCGCG (Phosphate). DNA copy numbers were determined from standard curves obtained using DNA samples extracted from HeLa R7 Neo cells, which were estimated to contain 1.24 ± 0.03 copies of proviral cDNA per cell [20]. Results were normalized by dividing by the number of cells, using the Light Cycler control kit according to the manufacturer's instructions (Roche Diagnostics).

### Western blot analysis

Cells were lysed in lysis buffer (20 mM Tris pH 7.5, 50 mM NaCl, 2 mM EDTA, 1% Triton X-100). The protein concentration of the extract was determined by Bradford assay, using the Coomassie Protein Assay Reagent (Pierce). Equal amounts of protein were loaded into each well of a polyacrylamide gel, subjected to SDS-PAGE and transferred to PVDF membranes for immunoblotting. Membranes were exposed to X-ray films or revealed by the Fuji LAS-3000 video acquisition device.

### Antibodies

Anti-GST-TBPEu^3+ ^and anti-HisXL665 antibodies were purchased from Cisbio Intl. Rabbit anti-HuR antibody was obtained from Upstate. Goat anti-actin, mouse monoclonal anti-HuR and rabbit anti-His antibodies were obtained from Santa Cruz Biotechnology. Rabbit polyclonal anti-p24, mouse monoclonal EVA3019 anti-HIV-1 RT and rabbit anti-HIV-1 p24 antibodies were obtained from the NIBSC Centralised Facility for AIDS Reagents supported by the EU program EVA/MRC (contract QLKZ-CT-1999-00609) and the UK Medical Research Council, and were kindly provided by Dr D. Helland and Dr A.M. Szilvay (anti-RT) and Dr G Reid (anti-p24). Mouse monoclonal anti-FLAG M2, rabbit polyclonal anti-FLAG, and mouse monoclonal anti-HA antibodies were obtained from Sigma. Horseradish peroxidase (HRP)-coupled anti-mouse, anti-rabbit and anti-goat secondary antibodies were obtained from Dako. Fluorescent secondary antibodies directed against rabbit FITC, rabbit Cy3, mouse FITC and mouse Cy3 were obtained from Jackson ImmunoResearch.

### Computational analysis

ARE-containing mRNA sequences were aligned, using the AlignX program of VectorNTI AdvanceTM software (Invitrogen). RNA secondary structures were determined, using the MFOLD program [22]. Accelrys Discovery Studio software was used to visualise the binding site of HuR on the RT heterodimer (PDB 1D 1HMI). Quantitative analysis of the siRNA silencing of HuR by Western blot was done with the Multi-Gauge software associated with the Fuji LAS-3000 video acquisition device.

### Immunoprecipitation assays

The protocol used to detect mRNAs bound to HuR has been described elsewhere [23,24]. HeLa cells (10^6 ^cells) were lysed in a lysis buffer (50 mM Tris pH 7.5; 150 mM NaCl, 1% Nonidet P40, 0.5% sodium deoxycholate). The supernatant was precleared with 2 μg of IgG1 (Santa Cruz Biotechnology) and 50 μl of protein G-agarose (Roche). The cleared supernatant was then incubated with 2 μg of mouse anti-HuR or mouse anti-HA antibody for 1 hour at 4°C. We then added 50 μl of protein G-agarose and incubated the mixture overnight at 4°C. Beads were washed five times in lysis buffer and treated with RNase-free DNaseI and proteinase K. RNA was extracted with phenol/chloroform, precipitated, and reverse-transcribed using MLV RT and random primers (Invitrogen). Precipitated mRNA was detected by qPCR, using the protocol and primers described by Lal *et al*. [23]. The primers used to detect Gag-Pol mRNA were the same as those used to detect the full-length HIV cDNA (late RT product).

## Results

### HuR is a cellular protein interacting with HIV-1 p66 reverse transcriptase

**Figure 1:**
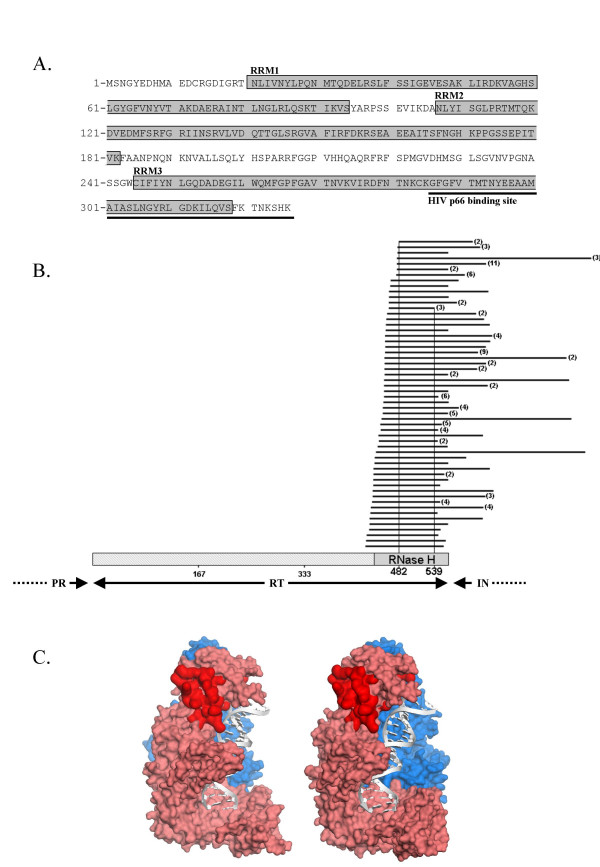
**Identification of HuR as a partner of HIV-1 p66 reverse transcriptase**. **A**. A yeast two-hybrid screen was carried out with HIV-1 RT-p66 as the bait, and a CEMC7 cDNA library as the prey. Amino-acid sequence of HuR and its predicted binding site to HIV-1 p66. RRM: RNA recognition motif. **B**. Alignment of the different fragments of HIV-1 interacting with HuR in the yeast two-hybrid rebound screen, using HuR as the bait and random fragments of HIV-1 YU-2 isolate as the prey. Numbers in brackets indicate the occurrence of each fragment. **C**. Mapping of the HuR interaction site on HIV-1 RT bound to a primer-template. Solvent accessible surface (probe radius 1.4 A) of the protein is represented in two different views (PDB 1D 1HMI) [53]. The p51 is shown in blue and p66 in pink. The DNA primer-template is represented in grey. The putative HuR binding site on p66 is represented in red.

We used a yeast two-hybrid screening system to identify cellular proteins able to interact with HIV-1 p66 reverse transcriptase. HIV-1 p66 fused to the LexA binding domain (LexA BD) was used as a bait to screen random primed cDNA libraries of CEMC7 lymphocytes, fused to the Gal4 activator domain. HuR fragments interacting with p66 HIV-1 RT were identified. All the fragments obtained contained the region of HuR between amino acids 286 and 326, which overlaps the third RNA recognition motif (RRM) in the C-terminal region of HuR (fig. 1A). This region constitutes the binding site of HIV-1 RT on HuR.

We assessed the specificity of HuR interaction with HIV-1 RT and mapped the HuR binding site on HIV-1 RT, by carrying out a yeast two-hybrid rebound screening, using HuR fused to LexA BD as the bait and a library of random fragments of HIV-1 DNA as the prey. This library of random HIV-1 DNA fragments was obtained from DNA sheared by nebulization, and then repaired and fused to Gal4 AD, as previously described [25]. All the random fragments of HIV-1 DNA that interacted with HuR included part of the RT sequence – the RNAse H region, in particular (Fig. 1B). No HIV-1 fragment interacting with HuR was found outside the RT sequence. The results of this rebound screen confirmed the specificity of the interaction between the two proteins, and allowed us to map the site of interaction with HuR between amino acids 482 and 539 in the C-terminal region of p66, corresponding to the domain with RNase H activity (fig. 1B).

Mapping of the predicted binding site for HuR on the RT heterodimer bound to a primer-template DNA revealed that it is freely accessible and extends to the vicinity of the primer-template. This observation leaves open the possibility of a simultaneous interaction of HuR with both RT and viral RNA (fig 1C).

### Purified GST-HuR and HIV-1 reverse transcriptase interact together in an *in vitro *assay

**Figure 2:**
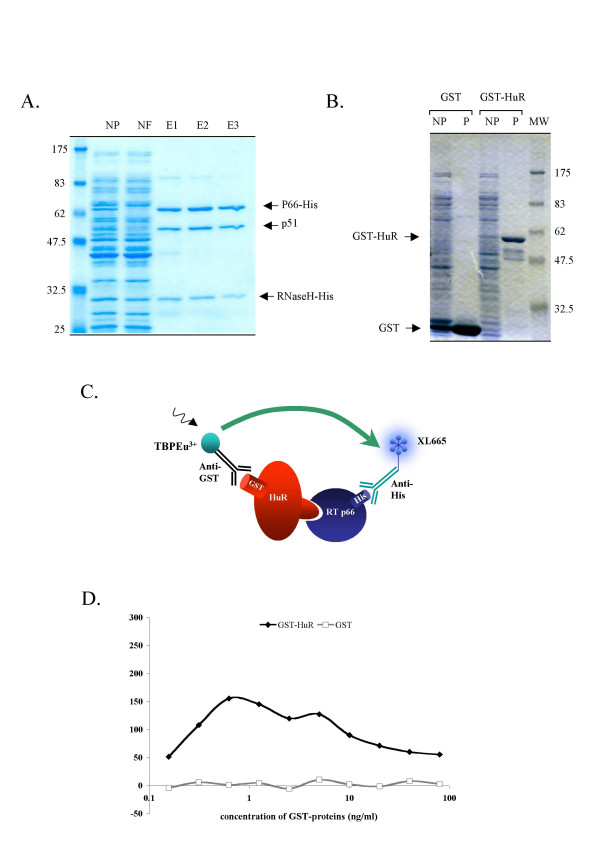
**The HIV-1 RT heterodimer interacts directly with GST-HuR**. **A**. Coomassie blue staining of the purified RT heterodimer. NP: non purified, NF: non fixed, E1–E3: elutions. **B**. Coomassie blue staining of the purified GST and GST-HuR. NP: non purified, P: purified. **C**. Schematic representation of our HTRF assay (adapted from Cisbio Intl.). Europium trisbipyridine cryptate (TBPEu^3+^) was coupled to anti-GST antibodies, acting as the FRET energy donor, following excitation at 337 nm. Cross-linked allophycocyanin (XL665) was coupled to anti-His antibodies acting as the FRET energy acceptor and emitting a sustained signal at 665 nm. **D**. Serial dilutions of purified GST-HuR or GST alone were incubated with a constant concentration of RT-His (10 ng/ml), in the presence of constant amounts of anti-His-XL665 and anti-GST-TBPEu^3+ ^antibodies. The Fret signal was measured after 24 hours of incubation at 4°C. These results are representative of those obtained in four independent experiments.

We produced and purified the recombinant proteins, to confirm the interaction between the two predicted partners *in vitro*. We used p6H-RT-PR, a vector allowing the simultaneous production of a C-ter 6xHis-tagged form of HIV-1 p66 reverse transcriptase together with the HIV-1 protease [16]. The products of C-ter 6xHis-tagged p66 cleavage by HIV-1 protease are untagged p51 and C-ter 6xHis-tagged RNaseH. The simultaneous production of cleaved and uncleaved p66 favors the formation of a well folded, fully functional p66/p51 RT heterodimer. Purified 6xHis-proteins were separated by reducing SDS-PAGE and stained with Coomassie blue to assess their purity (fig. 2A). Recombinant RT production was also checked by western blotting (data not shown). As expected, anti-RT monoclonal antibodies detected both RT chains, whereas anti-6xHis antibodies recognized only p66. As the affinity between p66 and p51 is strong, the detection of the p51 chain by Coomassie blue staining results from its coprecipitation with purified p66-His, rather than its binding to the affinity beads.

We also inserted the HuR gene into pGEX4T1, to produce a GST-HuR fusion protein. Purified GST-proteins were separated by SDS-gel electrophoresis and stained with Coomassie blue, to assess their purity (fig. 2B).

The purified recombinant p66-His and GST-HuR proteins were used in an HTRF interaction assay (reviewed in [26,27]). A schematic representation of the principle underlying this assay is shown in figure 2C. GST-HuR and C-ter 6xHis tagged RT-p66 are incubated with anti-GST antibodies conjugated with a fluorescence energy donor TBPEu3+, and anti-6His antibodies conjugated with a fluorescence energy acceptor XL665. Upon TBPEu^3+ ^excitation at 337 nm, a fluorescence resonance energy transfer signal emitted at 665 nm by the XL665 conjugate can be detected if an interaction occurs between the two recombinant proteins. The magnitude of this signal depends on the respective concentrations of the two interacting proteins.

Serial dilutions of the purified GST-HuR or GST alone were incubated for 24 hours at 4°C in the presence of constant amounts of antibodies against the 6xHis and GST tags, and a constant concentration of RT-His (20 ng/ml in total present in the reaction mixture, corresponding to about 10 ng/ml of p66-His, as evaluated by densitometry). As expected, a bell-shaped curve was obtained (fig. 2D). At lower concentrations, too little GST-HuR was present in the complex with RT-His and, at higher concentrations, some of the anti-GST antibodies were captured by the excess GST-HuR not associated with RT-His, thereby diminishing the signal. We obtained a signal with GST-HuR, but not with GST alone, consistent with a specific interaction. The two peaks obtained may result from the interaction of GST-HuR with both the full-length C-ter 6xHis p66 and the C-ter 6xHis RNaseH copurified on IMAC resin (fig. 2A). These results confirm that the RT-p66 and HuR recombinant proteins can interact *in vitro *and that this interaction is specific, as it does not take place with GST alone used as a control.

### HuR is important for the early steps of the HIV-1 replication cycle

**Figure 3:**
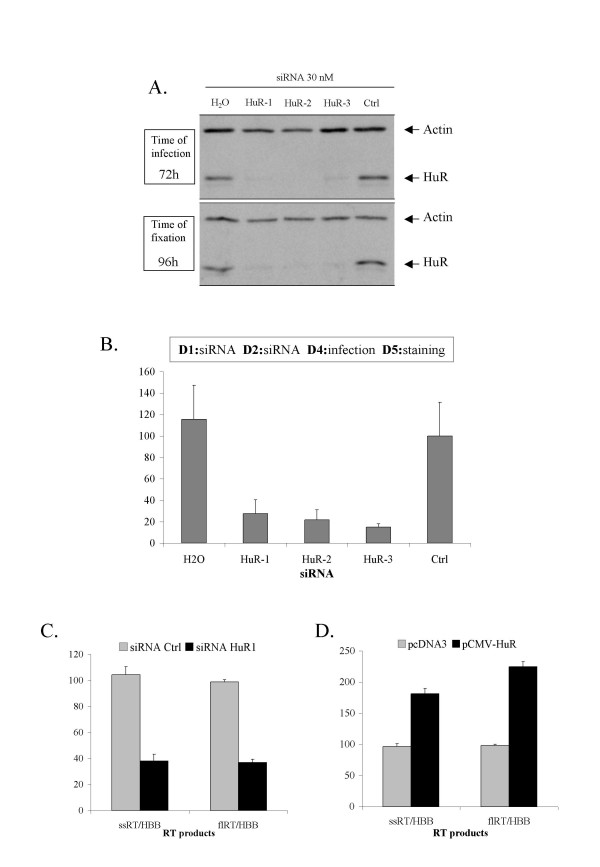
**HuR is involved in the early steps of HIV-1 replication cycle**. ***A. siRNA silencing of HuR revealed by Western blot***. HeLa P4.2 cells (CD4^+^, LTR-*LacZ*) were transfected with 30 nM of siRNAs directed against HuR (HuR1, HuR2, HuR3), H_2_O, or a non targeting siRNA (Ctrl). For each siRNA, five wells were infected with the HIV-1_NL4.3 _strain, 72 hours after transfection with the siRNA. The contents of one well were collected and lysed, to check that HuR expression was silenced at the time of infection (**A, upper panel**). ***B. Effect of HuR silencing on the infection of cells by wild type HIV-1***. 24 hours post-infection, infected cells were counted after the fixation and X-Gal staining of triplicate wells. The contents of one well were collected and lysed, to check that HuR expression was effectively silenced at the time of fixation (**A, lower panel**). The results presented are a compilation of six independent experiments, normalized as a function of the results obtained with the control siRNA (Ctrl). ***C. Effect of HuR silencing on HIV-1 reverse transcription***. HeLa cells were treated with siRNA HuR-1 or a non targeting siRNA (Ctrl), then infected with HIV-1ΔEnv-luciferase VSV-G pseudotyped viruses at an MOI of 1. Total DNA was extracted from the infected cells and RT products were quantified by quantitative real-time PCR, 16 hours after infection. ssRT: minus-strand strong stop DNA, flRT: full-length HIV DNA, HBB, human beta-globin. ***D. Effect of HuR overexpression on HIV-1 reverse transcription***. As in C, except that the cells were transfected with a vector allowing HuR overexpression (pCMV-HuR) or an empty vector (pcDNA3), before infection.

We evaluated the potential role of HuR in the HIV-1 replication cycle, using RNA interference techniques for gene silencing. We first monitored the early steps of the viral replication cycle, using an assay dependent on the correct entry, reverse transcription and integration of HIV into the cell genome. Reporter HeLa P4.2 cells (CD4+, LTR-*LacZ*, endogenous CXCR4) were independently transfected with three different siRNAs targeting different regions of the HuR mRNA, a negative control siRNA or no siRNA. Three days later, cells were infected with the X4 tropic strain HIV-1_NL4.3_. An aliquot of the transfected cells was lysed at the time of infection and HuR silencing was assessed by western blotting (fig. 3A, upper panel). A 90% decrease in HuR levels was observed. Cells were fixed 24 hours after infection, and stained with X-Gal, as previously described [19]. An aliquot of cells was collected, lysed and analyzed by western blotting. HuR knockdown was maintained throughout the experiment, as 90% silencing of HuR was still observed at the time of fixation (fig. 3A, lower panel). Tat-activated LTR was used for β-galactosidase production and the counting of successfully infected cells (fig. 3B). These results show significant impairment of the infection of HeLa P4.2 cells treated with the three different siRNAs. The similar levels of downregulation obtained with all three siRNAs, despite differences in the regions of the HuR mRNA targeted, and the similar phenotypic effects of these three siRNAs in our assay suggest that HuR may be involved in the early steps of the HIV-1 replication cycle.

We further assessed the importance of HuR in the early steps of HIV infection, by studying the reverse transcription products generated in infected cells in the presence and absence of HuR. We transfected HeLa cells with siRNA HuR1 or a control siRNA and infected them 48 hours later with non-replicative HIV-1ΔEnv-luciferase VSV-G pseudotyped viruses. The viral DNA produced by reverse transcriptase during this single cycle of infection were quantified by quantitative real-time PCR, using primers specific for early products (minus-strand, strong stop DNA) or late products (full-length DNA), as described in Materials and Methods. In cells treated with the HuR1 siRNA, the levels of both transcription products were much lower than those in cells treated with the control siRNA (fig. 3C). We also investigated the effects on reverse transcription of increasing HuR levels, by transfection with a vector allowing the overexpression of HuR (pCMV-HuR). In the presence of HuR overproduction, by contrast with what was observed with HuR silencing, both early and late products of reverse transcription were more abundant than in mock-transfected cells (fig. 3D). These results suggest a potential role for HuR in reverse transcription.

### HuR is not required for the post-integration steps of the HIV-1 replication cycle

**Figure 4:**
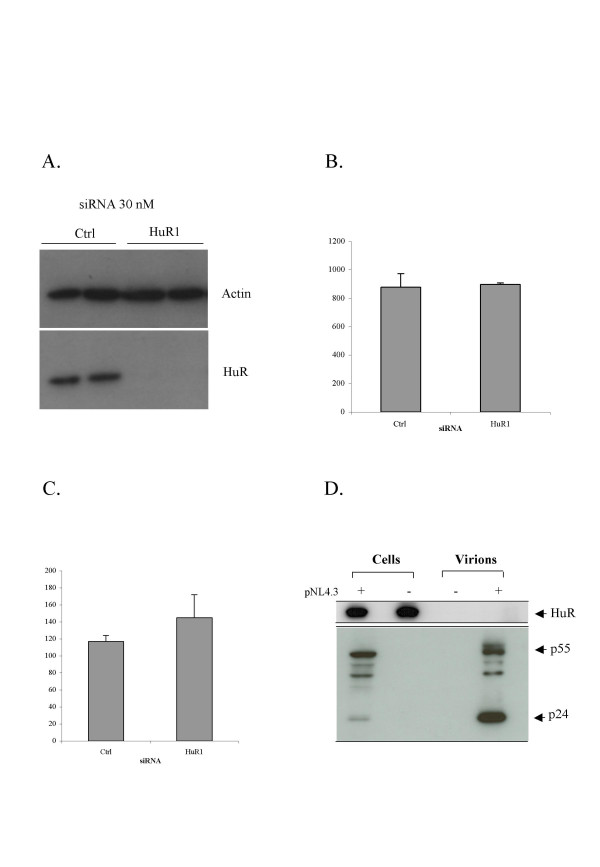
**HuR is not involved in the late steps of the HIV-1 replication cycle**. HeLa cells were transfected with an siRNA directed against HuR or a non-targeting siRNA (Ctrl). 24 hrs later, cells were transfected with HIV-1 provirus pNL4.3. **A**. Western blot confirming the silencing of HuR 48 hours after transfection with the siRNA. **B**. Quantification, by ELISA for Gag CA-p24 antigen, of the virions produced in the supernatant, 30 hours after transfection with pNL4.3. **C**. The virions produced in B were used to infect HeLa P4.2 cells (CD4^+^, LTR-*LacZ*). 24 hours post-infection, infected cells were fixed, stained with X-Gal and counted. **D**. 2 × 10^6 ^HEK293T cells were transfected with HIV-1 provirus pNL4.3. 48 hours later, the cell culture supernatant was collected, filtered and ultracentrifuged to collect the virions. Producer cells and virion pellets were lysed and analyzed by western blotting, to check their contents and HuR incorporation.

As the RNAse H domain found in our yeast two-hybrid screens is also a part of the Gag-Pol precursor, we investigated whether HuR also affected other steps of the viral replication cycle. We analyzed the impact of knocking down HuR levels in the producer cells. HeLa cells were treated with siRNA HuR1 or control siRNA. The cells were then transfected with the pNL4.3 provirus, making it possible to bypass the reverse transcription step. The silencing of HuR 48 hours after transfection with the HuR1 siRNA was assessed by western blotting (fig. 4A). No difference in virus production was detected between cells expressing and not expressing HuR, as identified by ELISA quantification of the Gag CA-p24 antigen in the supernatant (fig. 4B). We investigated whether HuR affected the infectivity of the viral particles, by using the supernatant of the cells in fig. 4B to infect HeLa P4.2 cells. No significant difference was observed in the number of infected cells (= infectious particles) (fig. 4C) or in the infectivity of these particles normalized on the basis of equal amounts of released p24 (data not shown). This result is consistent with the lack of detection of any HuR incorporated into viral particles produced from cells producing normal amounts of HuR (fig. 4D). Thus, HuR is unlikely to play a role in the late steps of the HIV-1 replication cycle, such as viral protein production, budding and maturation. Instead, it seems to act only in the target cell, following viral entry.

### Mutagenesis of a putative ARE sequence found in the HIV-1 genome

HuR has been reported to interact with ARE sequences found in the RNAs of several distantly related viruses, and is thought to be involved in their stabilization or expression [28-32]. We therefore investigated whether a similar phenomenon was also observed with HIV. We investigated in more detail the possible effects of HuR on the reverse transcription process, taking into account that HuR is generally considered to stabilize ARE-containing mRNAs, by checking HIV-1 RNAs for the presence of such ARE elements. Alignment analysis identified a sequence in HIV-1_NL4.3 _displaying significant similarity to known ARE sequences, and particularly to that of the prothymosin alpha (PTMA) mRNA (data not shown). An identical "hairpin" structure was predicted for both sequences (data not shown) [24]. The putative HIV-1 ARE sequence is situated in the coding sequence of *vif *and is remarkably conserved between HIV-1 isolates.

**Figure 5:**
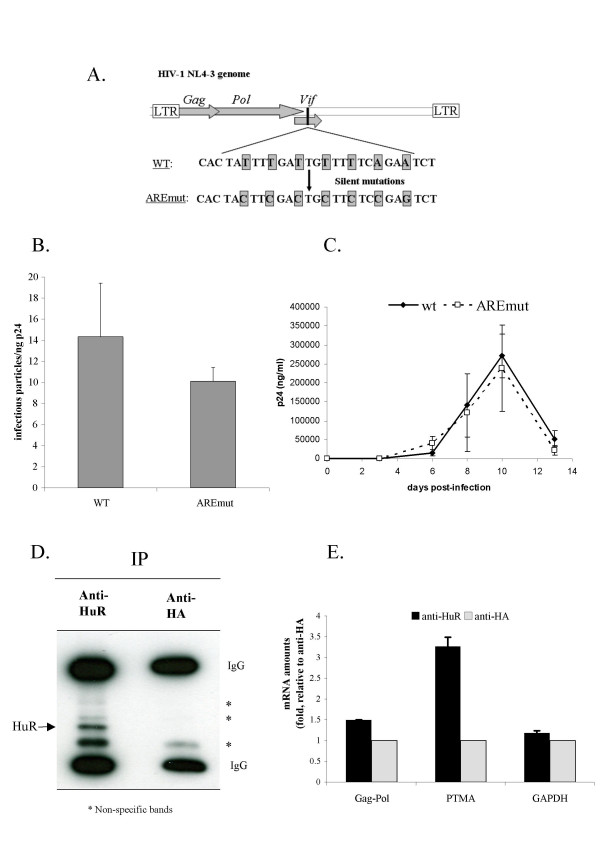
**HuR does not seem to bind to HIV-1 RNA**. **A**. Sequence of HIV WT ARE sequence and silent mutations introduced in the AREmut virus. **B**. HEK293T cells were transfected with WT or AREmut pNL4.3 proviruses. Quantification, by ELISA, of the virions produced in the cell culture supernatant, based on the detection of Gag CA-p24. **C**. Jurkat cells were infected with WT or AREmut NL4.3 viruses. Viral replication was monitored by ELISA quantification of HIV Gag CA-p24 antigen in the cell culture supernatant. **D**. Immunoprecipitation was carried out with anti-HuR antibodies or unrelated anti-HA antibodies. Western blot analysis, showing the immunoprecipitated protein. **E**. Coimmunoprecipitated mRNAs were detected by quantitative RT-PCR, using primers against HIV-1 *gag-pol*, *ptma *(as a positive control for HuR binding), and the housekeeping gene *gapdh *(as a negative control).

To verify the importance of this putative HIV-1 ARE sequence, we inserted several silent mutations into the coding sequence of pNL4.3, to deplete this region of U residues without affecting the amino acid sequence of *vif *(fig. 5A). HEK293T cells were transfected with this viral construct, to produce the mutated virus (AREmut). This virus was produced in similar amounts to the WT, although the viral particles were slightly less infectious (figure 5B). This mutated virus was used to infect Jurkat cells, and virus production was followed over time by quantifying HIV Gag CA-p24 antigen in the cell culture supernatant. No significant difference was observed between the replication kinetics of the WT and AREmut viruses (fig. 5C). These results are consistent with an absence of a role for the ARE motif or even with the presence of such a motif in this Vif sequence region of the HIV-1 RNA, although we cannot rule out the possibility that such a motif is present elsewhere in the HIV-1 genome.

### The role of HuR in HIV-1 reverse transcription does not seem to be mediated by binding to the HIV-1 RNA

We investigated whether HuR bound to a non typical class III ARE sequence elsewhere in the HIV-1 RNA, as for c-Jun [33], by determining the possible association of any HIV-1 RNA transcript with HuR, in an RNA-immunoprecipitation experiment using anti-HuR antibodies, as previously described [23]. We used HeLa R7 Neo cells stably infected with the HIV-1 neo Δ*env *virus [20], constituting a homogeneous population with similar levels of HIV-1 transcripts. RNA-immunoprecipitation experiments were carried out with anti-HuR antibodies or irrelevant anti-HA antibodies as the negative control. The immunoprecipitated proteins were detected by western blotting, showing the specific immunoprecipitation of HuR with anti-HuR antibodies and not with anti-HA antibodies (fig. 5D). As a positive control, PTMA mRNA, which is known to bind to HuR [23,24], was found associated with the immunoprecipitated HuR protein, as revealed by RT-PCR with the anti-HuR immunoprecipitate, using primers specific for PTMA mRNA (fig. 5E). The association of PTMA mRNA with HuR was specific, as the irrelevant immunoprecipitate obtained with anti-HA antibodies was not enriched in this RNA. The PTMA mRNAs precipitated with the anti-HuR antibody were 3.5 times more abundant than the negative control, the mRNA of the housekeeping gene *gapdh*. In contrast, the HIV-1 *Gag-Pol *transcript was not greatly enriched compared to PTMA mRNAs, since only a 1.5 folds increase was observed. This difference could be due to the relative abundance of the two mRNA species as well as a difference in the affinity of the interaction between HuR and the different mRNAs.

## Discussion

We performed a yeast two-hybrid screen, using the HIV-1 p66 RT subunit as the bait, to characterize cellular cofactors involved in the reverse transcription step of the HIV-1 replication cycle. We identified and validated an interaction between HIV-1 RT and the RNA-binding protein HuR. The HuR interaction site was mapped to the C-terminal part of the p66 RT subunit. This region, belonging to the RNase H domain, is freely accessible on the RT and extends to the vicinity of the primer-template. The p66 RT-HuR interaction was confirmed *in vitro *by an HTRF assay, suggesting that there was a direct interaction between HuR and p66 RT. However, since both HuR and RT are RNA binding proteins it could be possible that their interaction be mediated by RNA. Indeed, other interactions involving HuR have been shown to be RNA dependent, like the interaction between HuR and APOBEC3G [34]. HTRF assays conducted in the presence of RNAse did not allowed us to draw clear conclusions, since upon this treatment we obtained a slight and inconstant inhibition of the interaction signal (data not shown). Therefore, this question remains an open question that will need further investigations to be solved.

By silencing HuR expression with three different siRNAs targeting three different sites in the HuR mRNA sequence, we demonstrated that HuR expression was required for an optimal HIV-1 replication cycle and for both the early and late steps of reverse transcription, in particular. The enhancement of the reverse transcription reaction observed when HuR was overexpressed is consistent with these results. The absence of HuR affected wild type HIV-1, but also a non-replicative HIV-1ΔEnv-luciferase virus pseudotyped with the VSV-G envelope glycoprotein. As previously described, the entry pathways of these viruses are clearly differents [35]. While the wild type virus, bearing gp41/gp120, enters by fusion at the cell surface, VSV-G targets the virus to endocytosis and fusion in the endosomes. Although one cannot exclude this possibility, an effect of HuR on both entry pathways, in addition to its effect on reverse transcription, would be very unlikely. The effect of HuR seemed to be specific to the reverse transcription step in HIV target cells, as HuR silencing in HIV-1 producer cells had no effect on the production of viral particles or the infectivity of these newly released particles. Moreover, no incorporation of HuR into virions was observed, indicating that the HuR protein affecting reverse transcription was that present in the target cell, and not that in the producer cell.

The major role of HuR is to stabilize ARE-containing messenger RNAs (reviewed in [36,37]). This property of HuR seems to be related to its nucleocytoplasmic shuttling [8,38,39], following cellular stresses such as heat shock, exposure to UV light or infection [40]. Indeed, previous studies have reported the binding of HuR to the RNAs of various viruses, including HPV-1, HPV-16, *Herpesvirus saimiri *and HCV [28,31,32,41,42]. However, no interference of HuR with HIV-1 RNA has been reported in previous studies.

We identified a putative HuR binding motif, based on recent studies by Lopez de Silanes *et al*. [24]. We mutated this motif to disrupt the U-rich region. No effect on HIV replication was observed. Moreover, RNA-immunoprecipitation studies provided no evidence of an association between the HIV-1 RNA and HuR. This suggests that the mode of action of HuR in HIV-1 reverse transcription is based on its interaction with p66 RT rather than its interaction with the HIV-1 RNA. HuR plays a major role in stabilizing mRNAs, by binding to ARE elements, but previous studies have demonstrated protein-protein interactions involving HuR and playing an important role in the regulation of HuR activity [43-45]. One such interaction – with the RanGTP-binding nuclear transport receptor transportin 2 – was recently highlighted [46]. This interaction probably occurs in the cytoplasm, mediating the nuclear import of HuR. This interaction is optimal in the absence of RNA bound to HuR, suggesting that HuR is imported into the nucleus only when not bound to mRNA. The nucleocytoplasmic shuttling of HuR that seems to be responsible for mRNA stabilization was observed by Wang *et al*. upon T-cell activation, following the engagement of the integrin leukocyte function-associated molecule-1 (LFA-1) [10]. Several groups have previously reported the importance of LFA-1 for HIV infection and transmission to T cells [47-51]. As activated T cells are the preferred target cells for HIV infection, whereas unactivated T cells are very poorly infected by HIV, it is tempting to speculate that an absence of nucleocytoplasmic shuttling of HuR in unactivated T cells is correlated with the refractory state of these cells to HIV infection, together with other important recently discovered factors, such as the low molecular weight form of APOBEC 3G in these cells [52]. HuR has also been found in stress granules [9], together with APOBEC 3G [34], and is now considered to be a marker of these bodies. Is the ability of HuR to bind to p66 RT, positively affecting the reverse transcription of HIV-1 related to the nucleocytoplasmic shuttling property of HuR? Further work will be required to answer this important question.

In conclusion, we have identified a new cellular partner of HIV-1 reverse transcriptase: HuR. By modulating HuR levels, we were able to affect the infection of cells by HIV. However, the mechanism by which HuR influences the reverse transcription process remains to be elucidated.

## Abbreviations

HIV-1: human immunodeficiency virus type 1; RT: reverse transcriptase; siRNA: short interfering RNA; MOI: multiplicity of infection; GST: glutathione S-transferase; WT: wild type; HTRF: homogenous time-resolved fluorescence assay; VSV-G: vesicular stomatitis virus glycoprotein.

## Authors' contributions

JL designed and performed the siRNA experiments for analysis of infection, viral production and infectivity, produced the RT proteins, performed the HTRF experiments, and wrote the manuscript, PMP constructed and produced GST-HuR protein and the ARE-mutant, TB designed the HTRF experiments, EE mapped the HuR binding site on RT, JCR performed the yeast two-hybrid screening, RB conceived the study, participated to data analysis and contributed to the writing of the manuscript, LXL designed and performed the siRNA experiments for analysis of RT products by qPCR, analysed HuR incorporation into viral particles and its interaction with HIV-1 mRNA, and contributed to the writing of the manuscript. All authors read and approved the final manuscript.
